# Association between serum cotinine and muscle mass: The mediating role of systemic inflammation in NHANES 2013–2018

**DOI:** 10.18332/tid/231156

**Published:** 2026-10-31

**Authors:** Qingping Zheng, Jing Chen, Huanmin Zheng, Hao Feng, Qinyi You, Zhi Chen

**Affiliations:** 1 Department of Oncology, Laibin People’s Hospital, Laibin, China; 2 Department of Ophthalmology, Fuzhou University Affiliated Provincial Hospital, Fuzhou, China; 3 Department of Medicine and Nursing, Sanming Medical and Polytechnic Vocational College, Sanming, China; 4 Department of Orthopedics, Laibin People’s Hospital, Laibin, China; 5 Department of Geriatrics, Fujian Institute of Geriatrics, Fujian Key Laboratory of Vascular Aging, Fujian Clinical Research Center for Senile Vascular Aging and Brain Aging, Fujian Medical University Union Hospital, Fuzhou, China; 6 Department of Orthopedics Sports Injury Division, Fujian Medical University Union Hospital, Fuzhou, China

**Keywords:** serum cotinine, smoking, sarcopenia, mediation analysis, systemic inflammation

## Abstract

**INTRODUCTION:**

Smoking is a modifiable risk factor for muscle loss, but the underlying mechanisms are not fully understood. This study investigated the association between serum cotinine and appendicular skeletal muscle mass index (ASMI) and the mediating role of the systemic inflammation response index (SIRI).

**METHODS:**

Data from NHANES 2013–2018 were used for secondary analysis. A total of 4174 adults with complete records of cotinine and data for calculating ASMI and SIRI were included. Weighted multiple linear regression assessed associations of cotinine with ASMI and SIRI, and univariate analysis was performed to screen relevant variables. Mediation analysis with SIRI as the mediator subsequently quantified the indirect effect. All analyses were performed after adjustment for potential confounders.

**RESULTS:**

In females, ASMI was significantly lower in the high‑cotinine group than in the low‑cotinine group; no significant difference was observed between the two groups in males. Univariate analysis demonstrated significant negative correlations of cotinine and SIRI with ASMI. Across all weighted regression models, cotinine was negatively associated with ASMI (fully adjusted, β= ‑0.000; 95% CI: ‑0.000 – ‑0.000) and positively associated with SIRI (fully adjusted, β=0.001; 95% CI: 0.001–0.001). In the fully adjusted model, SIRI was negatively associated with ASMI (β= ‑0.003; 95% CI: ‑0.005 – ‑0.001). Mediation analysis showed that SIRI partially mediated the cotinine‑ASMI relationship, with a proportion mediated of 14.170%.

**CONCLUSIONS:**

Elevated cotinine levels are significantly associated with decreased muscle mass, and this association is partially mediated by systemic inflammation.

## INTRODUCTION

Sarcopenia is a muscle disorder that primarily affects older adults, characterized by declines in muscle mass, strength, and physical function. Data from a survey of 76151 communitydwelling older adults indicate a sarcopenia prevalence of 16.7%^1^. The figure rises to 65.9% among nursing home residents, and increases to 67.1% in individuals aged ≥80 years^2^. Sarcopenia not only leads to reduced physical function and lower quality of life in older adults but also significantly increases the risks of falls, fractures, hospitalization, and all‑cause mortality^3^. A longitudinal study of the older Chinese population showed that individuals with sarcopenia had a 2.3‑fold higher risk of death and a 1.8‑fold higher risk of hospitalization than those without sarcopenia^4^. Therefore, identifying modifiable risk factors is a priority for prevention.

As a modifiable risk factor, smoking has received increasing research attention for its impact on muscle mass. A review indicated that even in the absence of overt pulmonary pathology, smokers exhibit significantly lower muscle strength and fatigue resistance than non‑smokers. Mechanistically, cigarette smoke components may induce muscle loss by enhancing protein breakdown and inhibiting protein synthesis^5^. Exposure to cigarette smoke exacerbates local inflammatory responses and inhibits the activation of muscle satellite cells, thereby further impairing skeletal muscle structure and contractile function^6,7^. Among these mechanisms, systemic inflammation is a major pathophysiological response triggered by smoking and represents a pivotal link between smoking and muscle damage^8^.

Although an association between smoking and muscle loss is established^9^, the mechanisms underlying this relationship remain incompletely understood. Systemic inflammation response index (SIRI), as an emerging inflammatory biomarker, comprehensively reflects the body’s immune‑inflammatory status by integrating peripheral blood neutrophil, lymphocyte, and monocyte counts. In recent years, the diagnostic and prognostic value of SIRI in various diseases has been widely validated^10^. Therefore, this study examined whether SIRI mediates the cotinine–appendicular skeletal muscle mass index (ASMI) relationship and estimated the mediation proportion in the US population. The goal was to clarify one pathway through which smoking may affect muscle mass, rather than to provide a comprehensive mechanistic account.

## METHODS

### Data source

The NHANES is a continuous, cross‑sectional survey aimed at evaluating the health and nutritional status of the US population, including both children and adults. All survey protocols received approval from the National Center for Health Statistics (NCHS) Ethics Review Board, and written informed consent was obtained from every participant for the use of their data in research.

### Study population

This investigation utilized data from three NHANES cycles (2013–2018). Participants with complete records for cotinine, as well as necessary data to compute the ASMI and SIRI, were included. The exclusion criteria were: missing values for appendicular skeletal muscle mass (ASM) calculation (n=17573), missing data for fasting glucose (n=7337), insulin (n=125), or neutrophils (n=46); pregnancy (n=13); cancer diagnosis (n=113); missing weight data (n=18); and missing cotinine data (n=1). The participant selection process is illustrated in Figure 1. For each enrolled individual, we extracted and analyzed data on ASMI, SIRI, cotinine, and potential covariates.

**Figure 1:**
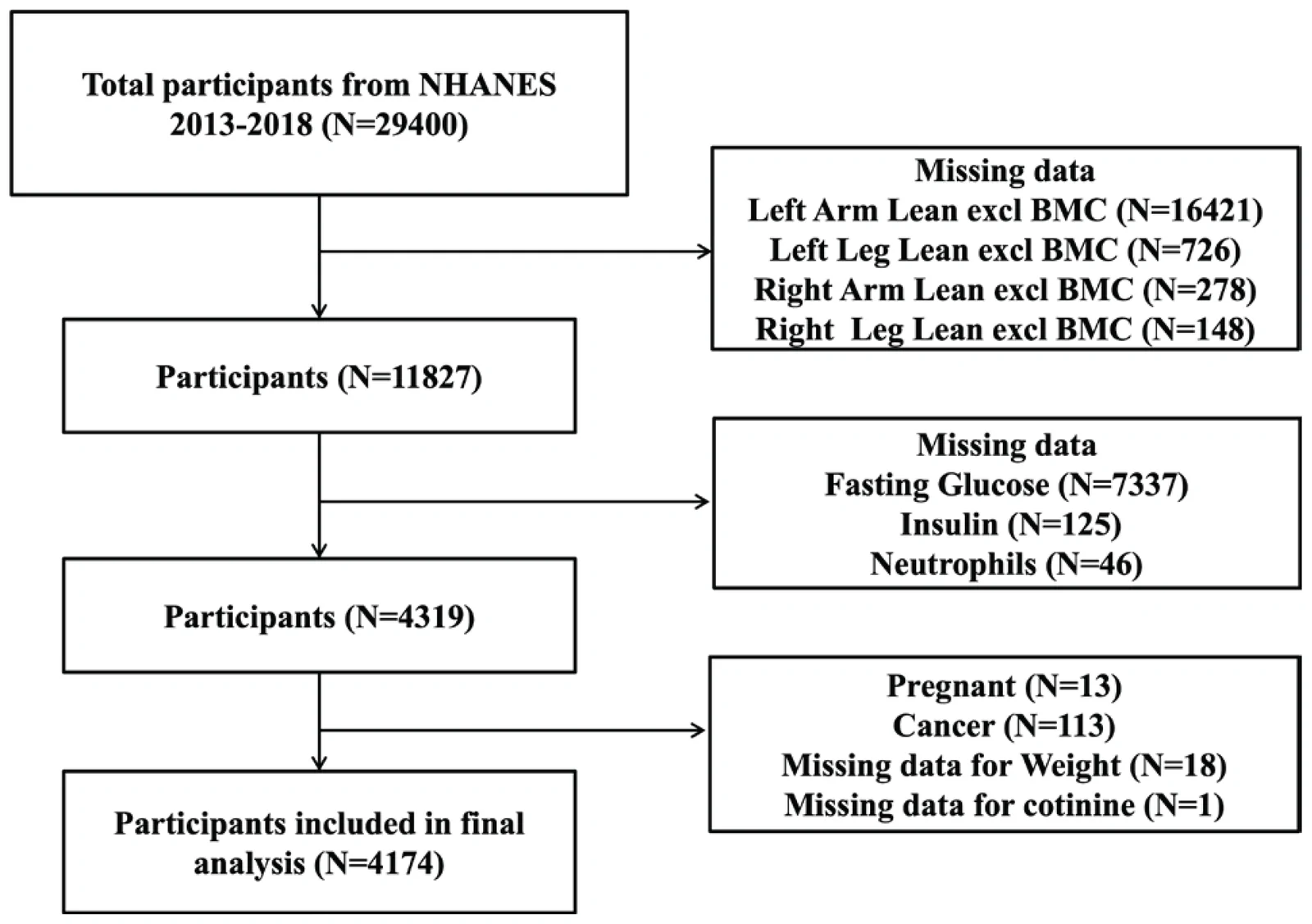
The flowchart of screening processes

### Study variables

*ASMI*

Dual‑energy X‑ray absorptiometry (DXA; Hologic, Inc., Bedford, Massachusetts) was employed to measure body composition, covering bone and soft tissue of the arms, legs, trunk, and head. ASM (kg) was derived as the sum of lean soft tissue mass in both arms and legs^9^. Muscle mass was then expressed as ASMI, defined as ASM divided by total body weight (ASM/Wt)^11^.

*SIRI*

The SIRI was computed as: (neutrophil count × monocyte count)/lymphocyte count^10^.

*Serum cotinine*

Serum cotinine levels (ng/mL) were measured using isotope‑dilution high‑performance liquid chromatography coupled with atmospheric‑pressure chemical ionization tandem mass spectrometry (ID HPLC–APCI MS/MS)^12^. Serum cotinine was analyzed as a continuous variable without transformation or categorization.

### Potential covariates

Covariates were chosen based on prior literature^9,12,13^. Demographic variables included age, sex (male, female), and race/ethnicity (Hispanic, non‑Hispanic White, non‑Hispanic Black, other races). Education level was categorized as below high school, high school, or college or higher. Hypertension and diabetes status were defined based on participants’ self-reported physician diagnosis from the questionnaire. Specifically, diabetes status was categorized according to responses to the question ‘Did the doctor tell you that you have diabetes’ with three response categories: ‘Yes’ (diabetes), ‘Borderline’ (borderline diabetes), and ‘No’ (no diabetes). Diabetes status was therefore coded as a three‑level categorical variable. Hypertension was defined as a ‘Yes’ response to the question ‘Ever told you had high blood pressure’ and was coded as a binary variable (yes, no). Fat mass from both arms and legs was summed to calculate total fat mass (FM), and the fat‑to‑muscle ratio (FMR) was derived as FM/ASM^9^. Fasting insulin and plasma glucose were measured using an immunoenzymometric assay on a TOSOH AIA‑900 chemistry analyzer. The HOMA‑IR was calculated as: [fasting insulin (µIU/mL) × fasting plasma glucose (FPG) (mmol/L)] / 22.5. Urine albumin was assessed by Fluorescein Immunoassay using a Sequoia‑Turner Digital Fluorometer, Model 450. Hemoglobin (g/dL) was measured via complete blood count with five‑part differential. Additional biochemical parameters: albumin (g/dL), total protein (g/dL), AST (U/L), ALT (U/L), uric acid (mg/dL), iron (µg/dL), lactate dehydrogenase (LDH, IU/L), and creatinine (mg/dL) were analyzed using a Beckman UniCel® DxC800 Synchron. Total cholesterol (mg/dL) was measured with a Roche/Hitachi Modular P Chemistry Analyzer, and glycohemoglobin (%) was determined using a Tosoh G8 Glycohemoglobin Analyzer. Other systemic inflammation indicators were calculated as follows: neutrophil‑monocyte to lymphocyte ratio (NMLR) = (neutrophil count + monocyte count)/lymphocyte count; systemic immune‑inflammation index (SII) = (platelet count × neutrophil count)/lymphocyte count^14^; and aggregate index of systemic inflammation (AISI) = (neutrophil count × monocyte count × platelet count)/lymphocyte count^15^. Comprehensive details regarding laboratory procedures, data processing, quality control, and analytic notes are available on the NHANES website.

### Statistical analysis

All statistical analyses were carried out using R software (version 3.4.3) and EmpowerStats (version 2.0). A two‑sided p<0.05 was considered statistically significant. To produce nationally representative estimates, NHANES sample weights were incorporated into all analyses to account for the complex survey design (stratification and clustering). Continuous variables were assessed for normality using the Shapiro‑Wilk test and visual inspection of histograms and Q‑Q plots. Normally distributed continuous variables were expressed as mean ± standard deviation (SD), while non‑normally distributed variables were summarized as median with interquartile range (IQR). Weighted linear regression models and weighted chi‑squared tests were applied for comparisons of continuous and categorical variables, respectively. Univariate linear regression was used to identify factors associated with ASMI. To examine the associations among serum cotinine, SIRI, and ASMI, three weighted multivariable linear regression models were constructed: Model 1 was unadjusted; Model 2 was adjusted for age, race, and education level; Model 3 was adjusted as for Model 2 plus diabetes, HBP, albumin, urine, ALT, AST, creatinine, iron, LDH, total protein, uric acid, hemoglobin, glycohemoglobin, and total cholesterol. Mediation analysis was performed with the ‘mediation’ package in R (version 4.2.2) to evaluate whether SIRI mediates the association between cotinine and ASMI. A significant mediating effect was determined by: 1) a significant indirect effect; 2) a significant total effect; and 3) a positive proportion mediated.

## RESULTS

Initially, a total of 29400 participants were identified. After applying the inclusion and exclusion criteria, 4174 participants were included in the final analysis. Participants were stratified by sex and cotinine level.

Among females, compared with the low‑cotinine group, those in the high‑cotinine group exhibited significantly higher FM (15.399 ± 6.323 vs 14.263 ± 5.694, p<0.001), FMR (0.811 ± 0.199 vs 0.784 ± 0.210, p=0.002), NMLR (2.205 ± 1.076 vs 2.088 ± 0.879, p=0.006), SIRI (1.132 ± 0.877 vs 0.932 ± 0.586, p<0.001), AISI (278.224 ± 247.030 vs 229.270 ± 169.342, p<0.001), ALT (22.615 ± 18.296 vs 20.980 ± 17.551, p=0.036), AST (25.546 ± 26.938 vs 22.692 ± 11.176, p=0.001), creatinine (0.807 ± 0.251 vs 0.746 ± 0.181, p<0.001), uric acid (5.118 ± 1.294 vs 5.000 ± 1.275, p=0.036), and hemoglobin (14.142 ± 1.566 vs 13.937 ± 1.361, p=0.001), as well as a higher prevalence of hypertension (18.839% vs 13.490%, p=0.002) and diabetes (5.333% vs 3.463%, p<0.001). Among females, participants in the high‑cotinine group had lower ASMI than those in the low‑cotinine group (0.249 ± 0.030 vs 0.252 ± 0.032, p=0.015).

Among males, compared with the low‑cotinine group, those in the high‑cotinine group exhibited significantly higher NMLR (2.307 ± 1.178 vs 2.167 ± 0.927, p=0.003), SIRI (1.224 ± 0.934 vs 1.041 ± 0.804, p<0.001), SII (504.340 ± 320.446 vs 464.173 ± 241.883, p=0.001), AISI (306.312 ± 262.630 vs 255.266 ± 206.708, p<0.001), creatinine (0.860 ± 0.296 vs 0.819 ± 0.530, p=0.030), and uric acid (5.702 ± 1.407 vs 5.517 ± 1.332, p=0.002), but lower albumin (4.244 ± 0.350 vs 4.291 ± 0.334, p=0.001) and total protein (7.152 ± 0.442 vs 7.213 ± 0.423, p=0.001). In males, there were no significant differences in FM, ASMI, or FMR between the high‑ and low‑cotinine groups (Table 1).

**Table 1:** Baseline characteristics of study participants, NHANES 2013–2018 (N=4174)

*Characteristics*	*Men*			*Women*		
	*Low cotinine*	*High cotinine*	*p*	*Low cotinine*	*High cotinine*	*p*
**Age** (years)	34.564 ± 13.421	35.263 ± 14.089	0.24849	35.184 ± 14.035	35.262 ± 13.777	0.89813
**Measurements**						
FM	10.916 ± 4.567	10.784 ± 5.174	0.53958	14.263 ± 5.694	15.399 ± 6.323	0.00002
ASMΙ	0.317 ± 0.035	0.319 ± 0.036	0.44306	0.252 ± 0.032	0.249 ± 0.030	0.01488
FMR	0.389 ± 0.144	0.390 ± 0.141	0.90547	0.784 ± 0.210	0.811 ± 0.199	0.00276
HOMA-IR	4.619 ± 6.402	4.860 ± 11.586	0.56183	2.694 ± 4.083	2.477 ± 2.531	0.14532
NMLR	2.167 ± 0.927	2.307 ± 1.178	0.00277	2.088 ± 0.879	2.205 ± 1.076	0.00616
SIRI	1.041 ± 0.804	1.224 ± 0.934	<0.00001	0.932 ± 0.586	1.132 ± 0.877	<0.00001
SII	464.173 ± 241.883	504.341 ± 320.446	0.00138	444.670 ± 230.243	466.638 ± 282.696	0.05034
AISI	255.266 ± 206.708	306.312 ± 262.630	<0.00001	229.270 ± 169.342	278.224 ± 247.030	<0.00001
Albumin, urine	29.281 ± 218.401	38.284 ± 247.822	0.38270	21.099 ± 95.460	29.513 ± 144.460	0.11406
ALT	27.630 ± 19.703	26.415 ± 18.092	0.14423	20.980 ± 17.551	22.615 ± 18.296	0.03657
Albumin	4.291 ± 0.334	4.244 ± 0.350	0.00170	4.366 ± 0.310	4.346 ± 0.337	0.15756
AST	25.463 ± 24.794	24.898 ± 16.036	0.53674	22.692 ± 11.176	25.546 ± 26.938	0.00139
Creatinine	0.819 ± 0.530	0.860 ± 0.296	0.02973	0.746 ± 0.181	0.807 ± 0.251	<0.00001
Iron	84.210 ± 35.064	84.262 ± 35.379	0.97328	88.719 ± 38.851	89.299 ± 41.698	0.74140
LDH	131.056 ± 28.851	132.482 ± 36.725	0.32897	126.797 ± 26.831	128.183 ± 30.236	0.26662
Total protein	7.213 ± 0.423	7.152 ± 0.442	0.00140	7.236 ± 0.405	7.208 ± 0.445	0.14077
Uric acid	5.517 ± 1.332	5.702 ± 1.407	0.00212	5.000 ± 1.275	5.118 ± 1.294	0.03568
Hemoglobin	14.302 ± 1.533	14.381 ± 1.586	0.25442	13.937 ± 1.361	14.142 ± 1.566	0.00135
Glycohemoglobin	5.614 ± 1.101	5.694 ± 1.103	0.09742	5.410 ± 0.865	5.430 ± 0.743	0.56542
Total cholesterol	183.802 ± 39.368	183.257 ± 39.846	0.75492	178.179 ± 41.313	174.990 ± 41.102	0.07631
Cotinine	0.016 ± 0.008	106.624 ± 151.922	<0.00001	0.016 ± 0.008	93.442 ± 141.560	<0.00001
**Race**			0.67128			0.51417
Mexican/Hispanic	20.101	20.644		18.859	17.342	
Non-Hispanic White	59.062	59.070		57.743	60.754	
Non-Hispanic Black	10.272	11.161		12.492	12.243	
Other races	10.566	9.125		10.906	9.660	
**Education level**			0.04447			0.40808
<High school	24.912	27.994		27.013	24.460	
High school	20.787	23.191		16.947	17.464	
>High school	54.300	48.816		56.041	58.076	
**Diabetes**			0.05970			0.00079
Yes	3.935	6.029		3.463	5.333	
No	94.171	92.632		95.489	91.760	
Borderline	1.894	1.339		1.048	2.907	
**High blood pressure**			0.17257			0.00228
Yes	25.344	28.095		13.490	18.839	
No	74.656	71.905		86.510	81.161	

Sampling weights were applied in all analyses. Mean ± SD for continuous variables: p-value was calculated by weighted linear regression model. Percent for categorical variables: p-value was calculated by weighted chi-squared test. Low cotinine group: serum cotinine concentrations in the lowest 50% of the study population; High cotinine group: serum cotinine concentrations in the highest 50%. The groups were defined by sorting serum cotinine concentrations in ascending order and dividing the study population into two groups with equal sample sizes. FM: fat mass. ASMI: ASM/Wt. FMR: fat-to-muscle mass ratio. HOMA-IR: Homeostatic Model Assessment of Insulin Resistance. NMLR: neutrophilmonocyte to lymphocyte ratio. SIRI: systemic inflammation response index. SII: systemic immune-inflammation index. AISI: aggregate index of systemic inflammation. LDH: lactate dehydrogenase.

### Univariate analyses

Univariate analyses (Table 2) revealed that older age (β= ‑0.001; 95% CI: ‑0.001 – ‑0.001, p<0.001), elevated cotinine (β= ‑0.000; 95% CI: ‑0.000 – ‑0.000, p<0.001), increased SIRI (β= ‑0.002; 95% CI: ‑0.004 – ‑0.000, p=0.035), and lower total cholesterol (β=0.000; 95% CI: 0.000–0.000, p=0.002) were each associated with lower ASMI in women.

**Table 2:** Results of univariate analysis for ASMI in female participants, NHANES 2013–2018 (N=4174)

*Variable*	*n (%)*	*ASMI* *β (95% CI) p*
**Age** (years), mean ± SD	33.069 ± 14.446	-0.001 (-0.001 – -0.001) <0.00001
**Race**		
Mexican/Hispanic	618 (29.359 )	0
Non-Hispanic White	681 (32.352 )	0.007 (0.004–0.011) 0.00008
Non-Hispanic Black	445 (21.140 )	0.022 (0.017–0.026) <0.00001
Other races	361 (17.150 )	0.014 (0.009–0.019) <0.00001
**Education level**		
<High school	750 (35.646 )	0
High school	370 (17.586 )	-0.014 (-0.018 – -0.010) <0.00001
>High school	984 (46.768 )	-0.007 (-0.010 – -0.004) 0.00002
**Diabetes**		
Yes	110 (5.228 )	0
No	1958 (93.061 )	0.020 (0.013–0.026) <0.00001
Borderline	36 (1.711 )	0.008 (-0.004–0.019) 0.17585
**High blood pressure**		
Yes	237 (13.852 )	0
No	1474 (86.148 )	0.003 (-0.001–0.007) 0.17123
**Measurements**	**Mean ± SD**	
Albumin, urine	25.236 ± 119.157	-0.000 (-0.000–0.000) 0.36020
ALT	21.029 ± 17.873	0.000 (-0.000–0.000) 0.31122
Albumin	4.370 ± 0.318	-0.002 (-0.006–0.002) 0.32603
AST	23.856 ± 23.558	0.000 (-0.000–0.000) 0.98872
Creatinine	0.765 ± 0.244	-0.001 (-0.007–0.005) 0.80644
Iron	88.935 ± 39.454	-0.000 (-0.000–0.000) 0.30566
LDH	128.119 ± 30.727	0.000 (-0.000–0.000) 0.25548
Total protein	7.222 ± 0.427	0.003 (-0.001–0.006) 0.11881
Uric acid	4.950 ± 1.268	0.001 (-0.000–0.002) 0.28329
Hemoglobin	14.013 ± 1.440	0.000 (-0.001–0.001) 0.81114
Cotinine	41.795 ± 107.318	-0.000 (-0.000 – -0.000) 0.00079
SIRI	0.989 ± 0.719	-0.002 (-0.004 – -0.000) 0.03510
Glycohemoglobin	5.385 ± 0.727	-0.001 (-0.002–0.001) 0.44819
Total cholesterol	175.149 ± 40.049	0.000 (0.000–0.000) 0.00215

All p-values were derived from weighted univariate linear regression models with ASMI as the dependent variable. Each row represents a separate univariate model. NHANES sampling weights were applied in all analyses.

### Association between cotinine and ASMI

Multivariable linear regression models were used to examine the relationship between cotinine and ASMI in women. In the fully adjusted model (Model 3), cotinine demonstrated a strong negative association with ASMI (β= ‑0.000; 95% CI: ‑0.000 – ‑0.000, p=0.003). Similar associations were observed in the unadjusted (Model 1) and partially adjusted model (Model 2) analyses (Table 3 and Figure 2).

**Table 3:** Weighted multivariable linear regression analysis examining the association between cotinine and ASMI among female participants, NHANES 2013–2018 (N=4174)

*Outcome*	*Model 1*		*Model 2*		*Model 3*	
	*β (95% CI)*	*p*	*β (95% CI)*	*p*	*β (95% CI)*	*p*
ASMI	-0.000 (-0.000 – -0.000)	0.00079	-0.000 (-0.000 – -0.000)	0.00552	-0.000 (-0.000 – -0.000)	0.00345

Sampling weights were applied in all analyses. Model 1: unadjusted. Model 2: age, race, and education level were adjusted. Model 3: adjusted as for Model 2 plus diabetes, HBP, albumin, urine, ALT, AST, creatinine, iron, LDH, total protein, uric acid, hemoglobin, glycohemoglobin, and total cholesterol.

**Figure 2:**
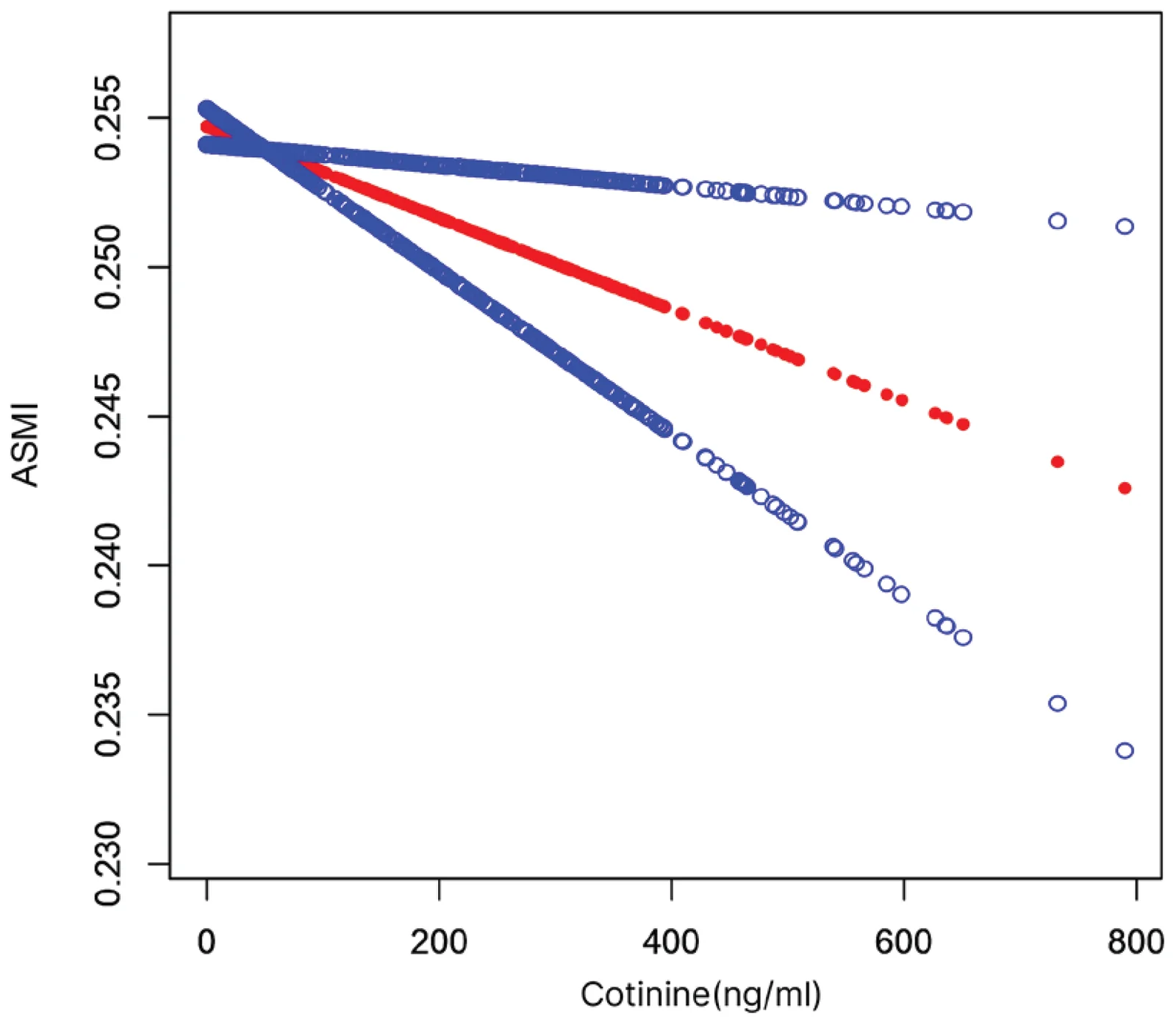
Weighted association between serum cotinine and ASMI among female participants, NHANES 2013–2018 (N=4174) (fully adjusted linear regression model)

### Association between SIRI and ASMI

Table 4 summarizes the associations between SIRI and ASMI in women across three multivariable linear regression models. In the fully adjusted model (Model 3), SIRI was significantly and negatively associated with ASMI (Model 3: β= ‑0.003; 95% CI: ‑0.005 – ‑0.001, p=0.006) (Figure 3).

**Table 4:** Weighted multivariable linear regression analysis examining the association between SIRI and ASMI among female participants, NHANES 2013–2018 (N=4174)

*Outcome*	*Model 1*		*Model 2*		*Model 3*	
	*β (95% CI)*	*p*	*β (95% CI)*	*p*	*β (95% CI)*	*p*
ASMI	-0.002 (-0.004 – -0.000)	0.03510	-0.001 (-0.003–0.000)	0.09209	-0.003 (-0.005 – -0.001)	0.00565

Sampling weights were applied in all analyses. Model 1: unadjusted. Model 2: age, race, and education level were adjusted. Model 3: adjusted as for Model 2 plus diabetes, HBP, albumin, urine, ALT, AST, creatinine, iron, LDH, total protein, uric acid, hemoglobin, glycohemoglobin, and total cholesterol.

**Figure 3:**
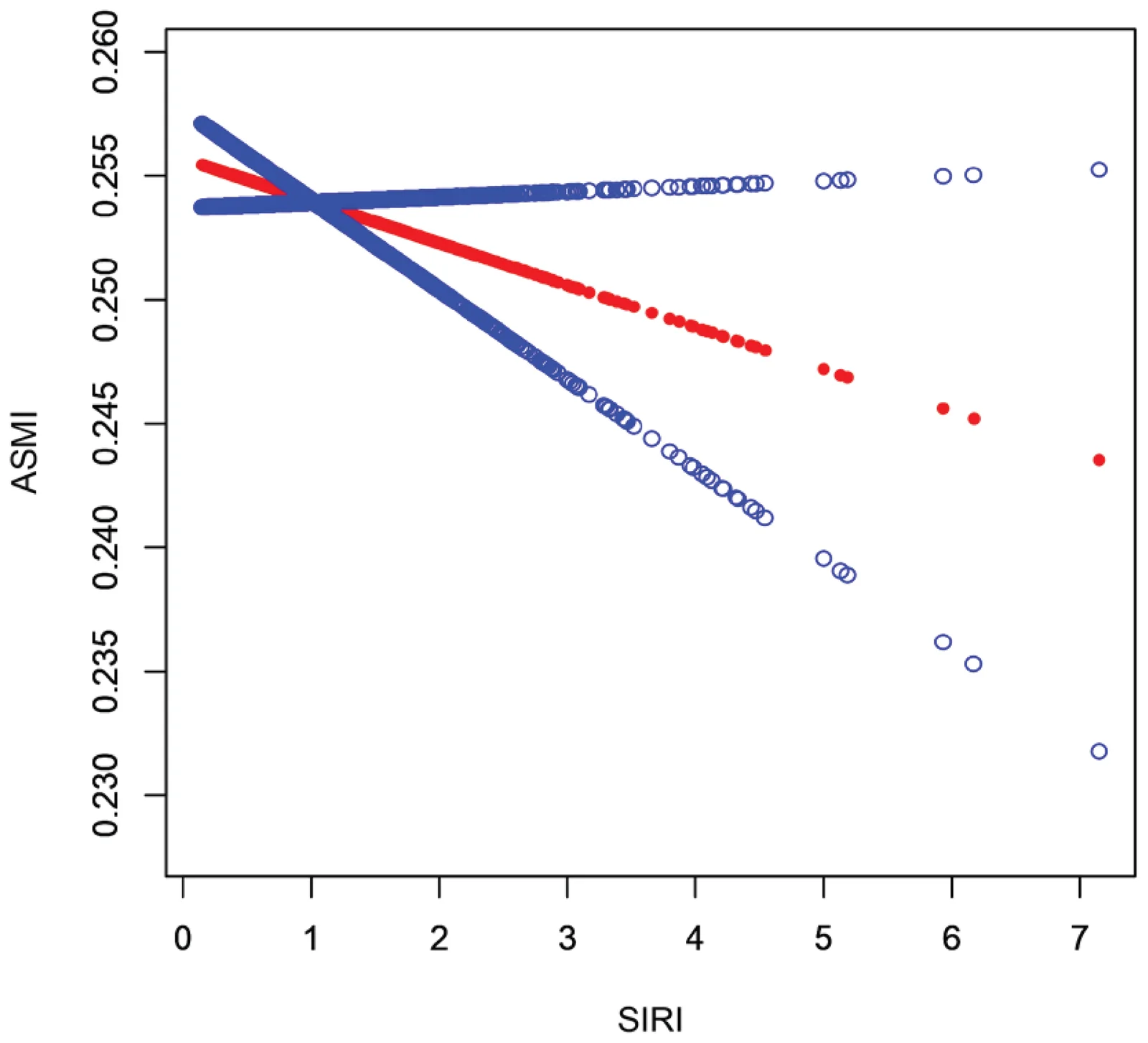
Weighted association between SIRI and ASMI among female participants, NHANES 2013–2018 (N=4174) (fully adjusted linear regression model)

### Association between cotinine and SIRI

Table 5 presents the results of relationships between cotinine and SIRI in women in all three multivariate linear regression models. In the fully adjusted model (Model 3), there was a significant positive association between cotinine and SIRI (Model 3: β=0.001; 95% CI: 0.001–0.001, p<0.001). Similar associations were observed in the unadjusted (Model 1) and partially adjusted model (Model 2) analyses (Table 5, Figure 4).

**Table 5:** Weighted multivariable linear regression analysis examining the association between cotinine and SIRI among female participants, NHANES 2013–2018 (N=4174)

*Outcome*	*Model 1*		*Model 2*		*Model 3*	
	*β (95% CI)*	*p*	*β (95% CI)*	*p*	*β (95% CI)*	*p*
Cotinine	0.001 (0.001–0.002)	<0.00001	0.001 (0.001–0.002)	<0.00001	0.001 (0.001–0.001)	<0.00001

Sampling weights were applied in all analyses. Model 1: unadjusted. Model 2: age, race, and education level were adjusted. Model 3: adjusted as for Model 2 plus diabetes, HBP, albumin, urine, ALT, AST, creatinine, iron, LDH, total protein, uric acid, hemoglobin, glycohemoglobin, and total cholesterol.

**Figure 4:**
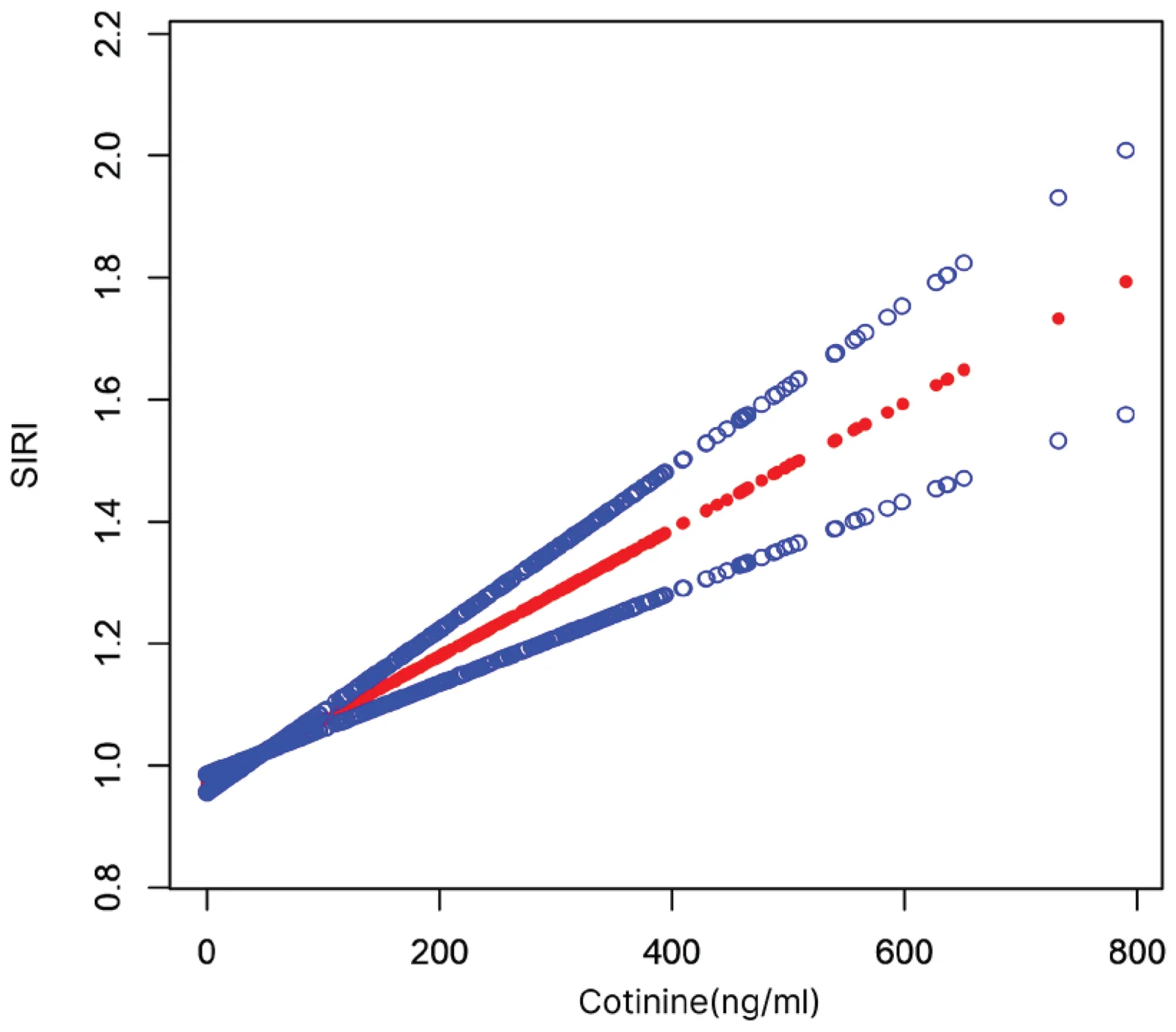
Weighted association between serum cotinine and SIRI among female participants, NHANES 2013–2018 (N=4174) (fully adjusted linear regression model)

### Mediating role of SIRI

Based on the fully adjusted model, mediation analysis demonstrated a significant indirect effect of cotinine on ASMI through SIRI (indirect effect= ‑0.000011, 95% CI: ‑0.000036 – ‑0.000006, p=0.004). The total effect (β= ‑0.000075) and direct effect (β= ‑0.000064) also remained significant. Approximately 14.170% of the association between cotinine and ASMI was mediated through SIRI (Table 6, Figure 5).

**Table 6:** Weighted causal mediation analysis examining the mediating role of SIRI in the association between cotinine and ASMI among female participants, NHANES 2013–2018 (N=4174)

*Characteristics*	*Estimate*	*95% CI*		*p*
		*Lower*	*Upper*	
Total effect	-0.000075	-0.000154	-0.000023	0.0060
Mediation effect	-0.000011	-0.000036	-0.000006	0.0040
Direct effect	-0.000064	-0.000130	-0.000005	0.0400
Proportion mediated	0.141695	0.066108	0.763755	0.0100

Age, race, education, diabetes, HBP, albumin, urine, ALT, AST, creatinine, iron, LDH, total protein, uric acid, hemoglobin, glycohemoglobin and total cholesterol were adjusted.

**Figure 5:**
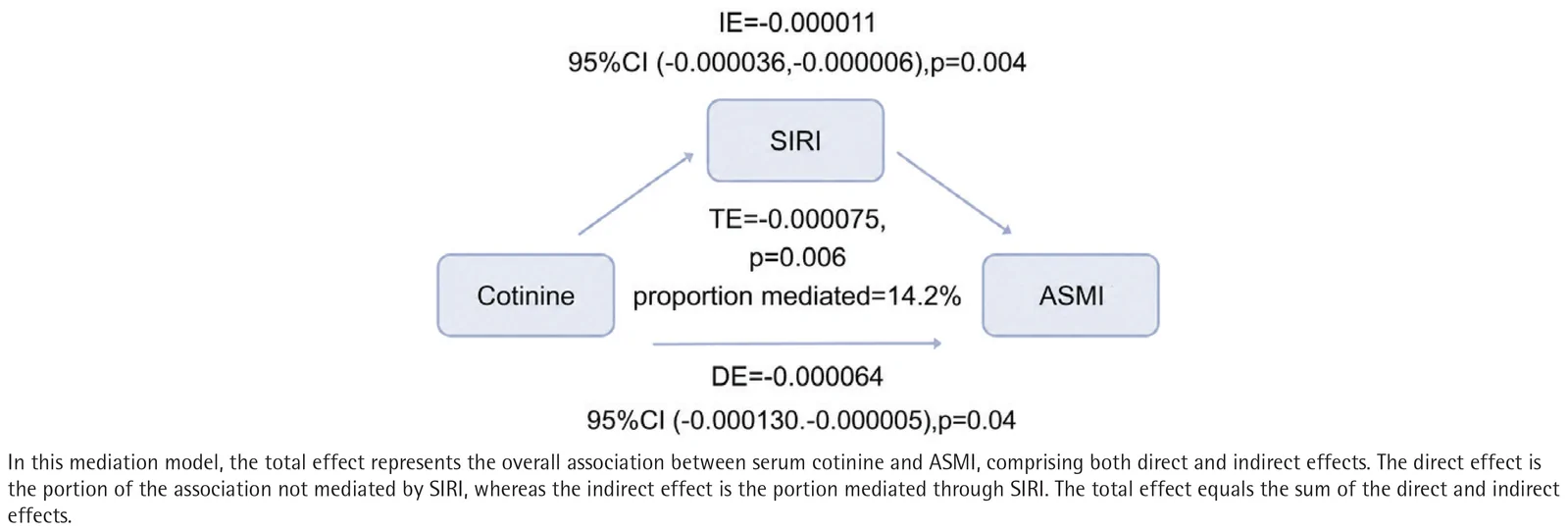
Mediation analysis of SIRI in the association between serum cotinine and ASMI among female participants, NHANES 2013–2018 (N=4174)

## DISCUSSION

In this cross‑sectional study of 4174 adults, we examined the association between cotinine and ASMI and further evaluated the mediating role of SIRI. Our findings demonstrated that cotinine was negatively associated with ASMI and positively associated with SIRI in women, whereas SIRI itself was negatively associated with ASMI. Mediation analysis revealed a significant indirect effect, with SIRI explaining approximately 14.170% of the total effect. These findings suggest that systemic inflammation may partially mediate the adverse effect of smoking exposure on skeletal muscle mass in women.

The observed association between serum cotinine and lower ASMI is consistent with previous evidence. A recent 16‑year cohort study from the Korean Genome and Epidemiology Study (n=8818) demonstrated a dose‑response relationship between cumulative smoking exposure and sarcopenia incidence, with smokers showing an approximately 28% higher risk than nonsmokers^16^. Moreover, a Mendelian randomization study by Sha et al.^17^ confirmed a causal relationship between genetically determined smoking and elevated sarcopenia risk, as well as a significant association with reduced appendicular lean mass. These findings suggest not only an association but also a potential causal link between smoking exposure and muscle mass loss. Nevertheless, the underlying mechanisms by which smoking contributes to muscle atrophy remain largely unexplored.

Our study further demonstrated that SIRI partially mediated the negative association between cotinine and ASMI, supporting the role of systemic inflammation in smoking‑related muscle loss. To our knowledge, this is the first large‑scale population study to quantify the mediating effect of systemic inflammation in this context. SIRI was significantly negatively associated with ASMI, consistent with previous evidence that proinflammatory cytokines promote muscle degradation^18,19^. Moreover, we observed a significant positive association between cotinine and SIRI, in line with basic research showing that cigarette smoke extract activates NFκB and increases proinflammatory cytokines^20^, and with clinical studies indicating that smoking history correlates with monocyte count, a component of SIRI^21^.

Although most previous studies support a negative impact of smoking on muscle mass, findings in the literature are not entirely consistent. Two large cross-sectional analyses of NHANES data demonstrated that higher serum cotinine was independently associated with decreased appendicular skeletal muscle mass index (ASMI), which is consistent with our findings^12,22^. In contrast, a cross‑sectional study using CT‑derived psoas muscle index (PMI) reported no significant differences in PMI or sarcopenia prevalence among active smokers, passive smokers, and non‑smokers^23^. Several methodological differences may account for these discrepancies. First, the assessment of smoking exposure differed substantially: our study and the NHANES‑based studies used serum cotinine as an objective biomarker, whereas the PMI study relied on self‑reported smoking status, which is susceptible to recall bias and misclassification. Second, the outcome measures varied: we quantified appendicular skeletal muscle mass using dual‑energy X‑ray absorptiometry (DXA), while the PMI study measured only the psoas muscle at the L3 level on CT, which may be less sensitive to global muscle loss. Third, the PMI study’s relatively small sample size (n=261) may have limited its statistical power to detect modest associations, whereas our study and the NHANES analyses included substantially larger populations. Fourth, differences in sarcopenia definitions – ranging from sample‑derived PMI cutoffs to standardized consensus criteria – may also contribute to the inconsistent results. Finally, variations in population characteristics, including age distribution, comorbidity burden, and racial composition, warrant consideration when interpreting these divergent findings.

Mechanistically, smoking activates TLR4 and triggers NLRP3 inflammasomemediated pyroptosis, releasing proinflammatory cytokines^20^, which in turn promote muscle protein breakdown via the ubiquitinproteasome system and impair satellite cell function^18,24^. Barreiro et al.^6^ found that cigarette smoke can directly induce oxidative modifications in muscle proteins, and that this oxidative damage occurs even before typical pathological changes appear in the lungs. Additionally, smoking indirectly impairs muscle health by lowering vitamin D levels, reducing estrogen activity, activating the aryl hydrocarbon receptor (AHR), and inducing mitochondrial dysfunction^25^.

Our subgroup analysis revealed that, among women, ASMI was significantly lower in the high‑cotinine group than in the low‑cotinine group, whereas no significant difference was observed in men. This finding suggests that the association between cotinine and ASMI differs by sex. Estrogen may protect muscle by promoting satellite cell activation and curbing inflammation; smoking can reduce estrogen activity via 2hydroxylation^26^, potentially making female smokers more vulnerable. In addition, because women have lower baseline muscle mass, a given absolute loss corresponds to a larger relative decline. Although we adjusted for potential confounders, future studies should further investigate the role of sex in the relationship between smoking and muscle loss.

### Strengths and limitations

Several limitations should be acknowledged. First, although we constructed a mediation model grounded in sound pathophysiological theory and statistically tested the mediating effect of SIRI, the cross‑sectional design of NHANES precludes determination of the temporal sequence among smoking exposure (cotinine), the mediator (SIRI), and the outcome (ASMI). The possibility of reverse causality or bidirectional relationships cannot be completely ruled out. Second, serum cotinine reflects recent exposure, and cotinine, SIRI, and ASMI were all measured at a single time point, capturing only a snapshot and not long‑term exposure or dynamic changes. Single measurements are prone to biological variability and measurement error, which may attenuate associations. Concurrent measurement of mediator and outcome limits causal inference regarding their temporal order. Additionally, source-specific exposure (e.g. e‑cigarettes, secondhand smoke) cannot be distinguished. Third, although we adjusted for multiple covariates, residual confounding from unmeasured factors, such as diet, physical activity, alcohol consumption, medication use, and socioeconomic status, may still exist. Fourth, hypertension and diabetes were self‑reported, which may have introduced recall bias. Participants may have inaccurately recalled or under‑reported their diagnoses, particularly those with undiagnosed or borderline conditions, leading to potential misclassification. Fifth, excluding participants with missing data may introduce selection bias if missingness was not completely at random, and analyzing only complete cases may limit the generalizability of our findings to the broader population. Sixth, various adjustment methods have been employed to quantify muscle mass, such as ASM/height‑squared, ASM/weight, and ASM/BMI. It has been suggested that different adjustment methods may yield different findings regarding the association between muscle mass and clinical outcomes. Therefore, an urgent need for consensus exists to standardize the adjustment methodology.

Despite these limitations, our findings carry important clinical and public health implications. Serum cotinine serves as an objective biomarker of smoking exposure, avoiding recall bias associated with selfreported history. SIRI is calculated from routine blood test parameters, requiring no additional cost and offering good clinical feasibility. From an intervention perspective, our study suggests that smoking cessation and anti‑inflammatory interventions may have synergistic effects in preventing smoking‑related muscle loss. The efficacy of these strategies, however, requires validation through prospective intervention studies.

## CONCLUSIONS

In the NHANES 2013–2018 population, smoking exposure was independently associated with reduced appendicular skeletal muscle mass index (ASMI). Systemic inflammation partially mediated the negative association between serum cotinine levels and ASMI, suggesting that smoking may contribute to muscle loss at least in part through the induction of inflammatory pathways. However, given the cross-sectional design, these findings should be interpreted as associations rather than evidence of causal relationships. Prospective studies and mechanistic investigations are warranted to confirm the role of smoking in musculoskeletal decline and to further support the clinical importance of smoking cessation for preserving muscle health.

## Data Availability

The data supporting this research are available from the following link (http://www.cdc.gov/nchs/nhanes/). Additional details or datasets generated during the current study are available from the corresponding author upon reasonable request. The authors confirm that NHANES data were accessed in compliance with the NCHS data access guidelines. This study utilized publicly available datasets, adhering to all regulations regarding data confidentiality and use. Data analysis and reporting were conducted in accordance with NHANES guidelines to ensure accuracy and integrity.
